# New data on the fauna of the water treaders (Hemiptera, Mesoveliidae) of Korea

**DOI:** 10.3897/BDJ.12.e136390

**Published:** 2024-10-14

**Authors:** Seokin Yang, Wonwoong Kim

**Affiliations:** 1 Department of Biological Sciences, Vanderbilt University, Nashville, United States of America Department of Biological Sciences, Vanderbilt University Nashville United States of America; 2 College of Literature, Science & the Arts, University of Michigan, Ann Arbor, United States of America College of Literature, Science & the Arts, University of Michigan Ann Arbor United States of America

## Abstract

**Background:**

The Family Mesoveliidae, also known as the water treaders, are predaceous semi-aquatic bugs with approximately 50 known species worldwide. In the Korean Peninsula, only two species were known prior to this study: *Mesoveliavittigera* Horváth, 1895 and *M.egorovi* Kanyukova, 1981.

**New information:**

A total of four species in two genera are recognised from Korea, including new distributional records of *Mesoveliathermalis* Horváth, 1915 and *Speoveliamaritima* Esaki, 1929. The record of *M.egorovi* Kanyukova, 1981 from Mt. Palgongsan, South Korea is doubtful. An identification key is provided to aid identification of the Korean species.

## Introduction

The water treaders (Hemiptera, Mesoveliidae) are a family of semi-aquatic bugs with approximately 50 known species around the globe (Damgaard et al. 2012, Schuh and Weirauch 2020). All known Mesoveliidae are predaceous, treading their habitats at high speed to forage on small aquatic/semi-aquatic arthropods (Zimmermann 1984, Damgaard 2008). Only three genera are known from the Palaearctic Region: *Mesovelia* (6 sp.), *Speovelia* (1 sp.) and *Nagisavelia* (1 sp.) (Andersen 1995, Watanabe et al. 2023). The cosmopolitan *Mesovelia* inhabit freshwater and brackish waterbodies with abundant floating vegetation coverage (Andersen and Polhemus 1980). On the other hand, *Speoveliamaritima* Esaki, 1929 and the recently-described *Nagisaveliahikarui* Watanabe, Nakajima & Hayashi, 2023 are known to inhabit caves or rocks near maritime environments and intertidal zones (Yuasa 1929, Nakajima et al. 2020, Watanabe et al. 2023).

In East Asia, the fauna of Mesoveliidae are well reported especially in Russia and Japan. In Russia, two species of the genus *Mesovelia* Mulsant & Rey, 1852 were described new to science (Kerzhner 1977, Kanyukova 1981) and comprehensive keys were given for the fauna of the Russian Far East (Kanyukova 1988). In Japan, two genera and species were newly described (Esaki 1929, Watanabe et al. 2023) and regional distribution and identification keys were provided by various authors (Miyamoto 1964, Miyamoto and Hayashi 1998, Hayashi and Miyamoto 2018, Nakajima et al. 2020).

In the Korean Peninsula, two species, *M.vittigera* Horváth, 1895 and *M.egorovi* Kanyukova, 1981, are known to date (Kwon et al. 2001, Korean Society of Applied Entomology and The Entomological Society of Korea 2021). However, the information on each species is generally scarce, except for brief collection records in taxonomic treatments or regional faunistic surveys. In addition, the Korean record of *M.egorovi* is currently questioned by several authors (Aukema et al. 2013, Kanyukova and Egorov 2022). To clarify the existing fauna of Mesoveliidae of Korea, we conducted a systematic survey in various regions of South Korea representing diverse climatic and geographical conditions. In total, four species in two genera are recognised, with two species reported from Korea for the first time.

## Materials and methods

The study is entirely based on materials that were collected during April to August 2024 in South Korea. Survey sites were selected to include wide ranges of ecotopes (e.g. pond and reservoir, rice paddy, marsh and brackish water) that represent putative habitats for Mesoveliidae.

Most samples were collected by sieving the surface-floating vegetation with a net and metal strainer. For *S.maritima*, however, the specimens were collected by visually inspecting the rocky shorelines with an aspirator. Collected samples were photographed under a Nikon D7500 camera, equipped with a Nikon AF Micro Nikkor 60mm f/2.8D lens and 36mm Auto Extension Tube. Examined specimens are deposited in the Museum of Zoology, University of Michigan (UMMZ).

Identification of samples followed the works of Kanyukova (1988) for fauna of the Russian Far East and Watanabe et al. (2023) for the Japanese species. The terminology used in this study followed that of Andersen and Polhemus (2003).

The Korean provinces mentioned below are abbreviated as follows: **CB**—Chungcheongbuk-do; **CN**—Chungcheongnam-do; **GG**—Gyeonggi-do; **GB**—Gyeongsangbuk-do; **GN**—Gyeongsangnam-do; **GW**—Gangwon-do; **JB**—Jeollabuk-do; **JN**—Jeollanam-do; **JJ**—Jeju-do.

## Taxon treatments

### Mesovelia egorovi

Kanyukova, 1981

2771447F-E226-5E55-B565-23571B899C1F

#### Distribution

Korea? (GB) (Lee et al. 1994), Japan (Hokkaido, Honshu, Shikoku, Kyushu, Nansei Islands) (Miyamoto and Hayashi 1998, Noishiki 2020, Watanabe 2020, Ito and Nakajima 2021, Mariko et al. 2024), Russia (Far East: South Sakhalin, Primorsky Krai) (Kanyukova 1988, Kanyukova and Egorov 2022).

#### Notes

*Mesoveliaegorovi* was initially recorded from South Korea by Lee et al. (1994), based on an unspecified number of specimens from Mt. Palgongsan, Gyeongsangbuk-do Province. These specimens were apparently collected from a mountainous habitat, far from the coastline (see Kanyukova and Egorov (2022) for details). However, all known records of *M.egorovi* are restricted to salt marshes and estuaries from the Russian Far East (Kanyukova and Egorov 2022) and Hokkaido and Honshu in Japan (Miyamoto and Hayashi 1998, Watanabe 2020). No additional specimens or distributional records of *M.egorovi* have been reported in South Korea since its initial report. Based on this evidence, the distributional record of *M.egorovi* in South Korea is regarded as doubtful; similar suggestions were made by early authors (Aukema et al. 2013, Kanyukova and Egorov 2022). Collection efforts in salt marshes and lagoons in Goseong, Sokcho and Gangneung, along the north-eastern coastline in South Korea by the authors also failed to locate this species.

### Mesovelia thermalis

Horváth, 1915

25E34F64-2F10-55AC-A93C-3D6DEED9A348

#### Materials

- **Type status:** Other material. **Occurrence:** recordedBy: Seokin Yang & Wonwoong Kim; individualCount: 3; sex: 1♂, 2♀; lifeStage: adult; occurrenceID: 8DA93A8A-E9D3-50AA-B9B6-76CF9B650E96; **Taxon:** scientificName: *Mesoveliathermalis* Horváth, 1915; family: Mesoveliidae; genus: Mesovelia; specificEpithet: *thermalis*; scientificNameAuthorship: Horváth, 1915; **Location:** country: Republic of Korea; stateProvince: Gyeonggi-do; locality: Ilsan Lake Park (37.658551, 126.761916) Goyang-si; **Identification:** identifiedBy: Seokin Yang & Wonwoong Kim; **Event:** eventDate: 13.v.2024
- **Type status:** Other material. **Occurrence:** recordedBy: Seokin Yang & Wonwoong Kim; individualCount: 1; sex: 1♂; lifeStage: adult; occurrenceID: 7651E809-6C79-5D64-8CFA-28FA051E704B; **Taxon:** scientificName: *Mesoveliathermalis* Horváth, 1915; family: Mesoveliidae; genus: Mesovelia; specificEpithet: *thermalis*; scientificNameAuthorship: Horváth, 1915; **Location:** country: Republic of Korea; stateProvince: Gyeonggi-do; locality: Wangsong Reservoir (37.3153654, 126.9472773), Uiwang-si; **Identification:** identifiedBy: Seokin Yang & Wonwoong Kim; **Event:** eventDate: 23.v.2024
- **Type status:** Other material. **Occurrence:** recordedBy: Seokin Yang & Wonwoong Kim; individualCount: 53; sex: 22♂, 31♀; lifeStage: adult; occurrenceID: 4005017C-1021-5EE0-9FE0-3AAA910F32D1; **Taxon:** scientificName: *Mesoveliathermalis* Horváth, 1915; family: Mesoveliidae; genus: Mesovelia; specificEpithet: *thermalis*; scientificNameAuthorship: Horváth, 1915; **Location:** country: Republic of Korea; stateProvince: Seoul; locality: Nanji Wetland (37.572056, 126.868340), Mapo-gu; **Identification:** identifiedBy: Seokin Yang & Wonwoong Kim; **Event:** eventDate: 28.v.2024

#### Distribution

Widespread in the Palaearctic Region (Andersen 1995). In East Asia: Korea (GG, Seoul), China (north-western) (Jorigtoo and Qi 1996), Japan (Hokkaido, Honshu, Shikoku) (Nakajima et al. 2020), Russia (Far East: Primorsky Krai) (Kanyukova 1988).

#### Biology

**Figure 2.**
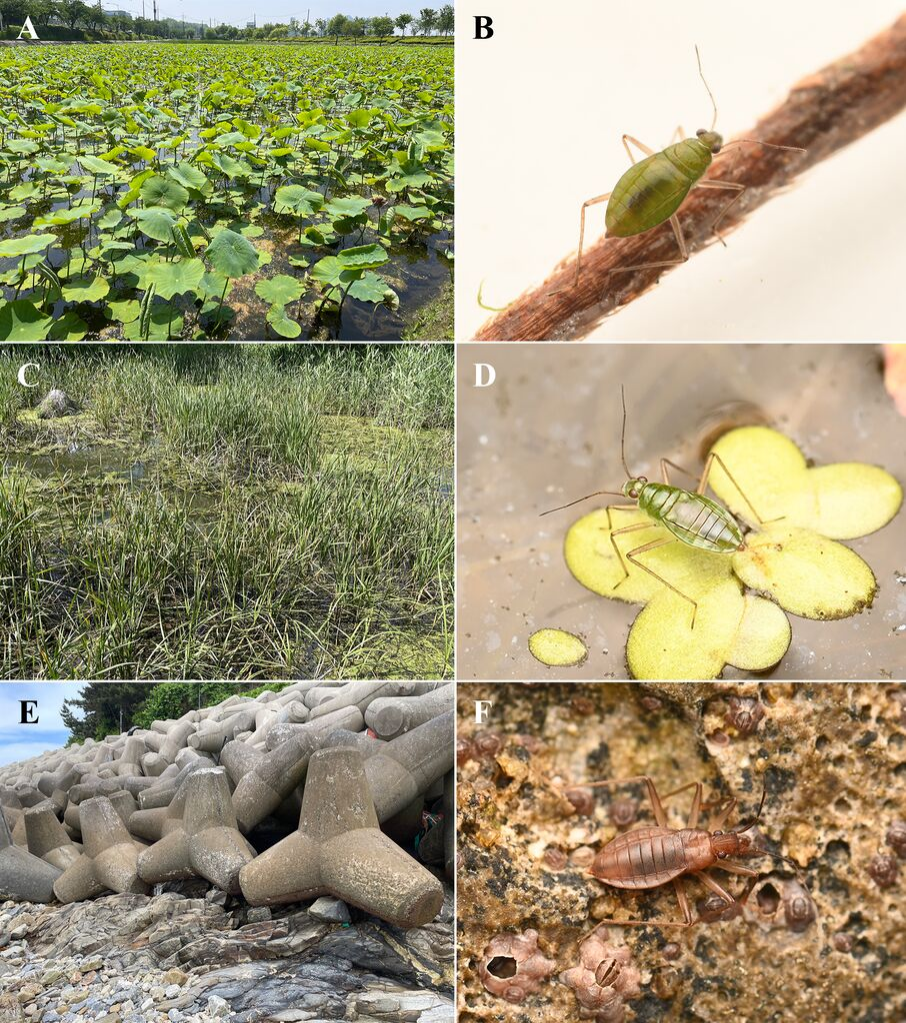
**Habitat and live individuals of Korean Mesoveliidae species. A** Habitat of *M.vittigera* - lotus cultivation area covered with floating vegetation; **B** *M.vittigera*, female, collected from this locality; **C** Habitat of *M.thermalis* - natural pond with abundant emergent and floating vegetation; **D** *M.thermalis*, female, collected from this locality; **E** Habitat of *S.maritima*, concrete tetrapod structures on the rocky shore; **F** *S.maritima*, female, collected from this locality.

Confirmed habitats of *M.thermalis* in Korea were freshwater bodies, both shaded and unshaded, with abundant emergent and floating vegetation (Fig. 2C). Observed specimens were mostly found on the floating vegetation during daytime. Some of the confirmed habitats were also occupied by *M.vittigera*; similar observations were reported by Berchi et al. (2016) in Romania.

#### Notes

New record for Korea.

##### Korean vernacular name

"Jul-mul-no-lin-jae" referring to the dark stripes along the thoracic and abdominal segments.

### Mesovelia vittigera

Horváth, 1895

BE633527-1305-5625-A7DA-E89588DEA14E

#### Materials

- **Type status:** Other material. **Occurrence:** recordedBy: Seokin Yang & Wonwoong Kim; individualCount: 5; sex: 4♂, 1♀; lifeStage: adult; occurrenceID: 5ED47349-A7AA-5E00-AA65-53427BF4139A; **Taxon:** scientificName: *Mesoveliavittigera* Horváth, 1895; family: Mesoveliidae; genus: Mesovelia; specificEpithet: *vittigera*; scientificNameAuthorship: Horváth, 1895; **Location:** country: Republic of Korea; stateProvince: Gyeonggi-do; locality: Ilsan Lake Park (37.661108, 126.762026), Goyang-si; **Identification:** identifiedBy: Seokin Yang & Wonwoong Kim; **Event:** eventDate: 21.v.2024
- **Type status:** Other material. **Occurrence:** recordedBy: Seokin Yang & Wonwoong Kim; individualCount: 7; sex: 3♂, 4♀ (1♂ macropterous); lifeStage: adult; occurrenceID: 6E830790-0AE6-50FA-B8C0-3C9496F584C0; **Taxon:** scientificName: *Mesoveliavittigera* Horváth, 1895; family: Mesoveliidae; genus: Mesovelia; specificEpithet: *vittigera*; scientificNameAuthorship: Horváth, 1895; **Location:** country: Republic of Korea; stateProvince: Gyeonggi-do; locality: Wangsong Reservoir (37.3153654, 126.9472773), Uiwang-si; **Identification:** identifiedBy: Seokin Yang & Wonwoong Kim; **Event:** eventDate: 23.v.2024
- **Type status:** Other material. **Occurrence:** recordedBy: Seokin Yang & Wonwoong Kim; individualCount: 6; sex: 5♂, 1♀; lifeStage: adult; occurrenceID: DE21B38F-1B34-5C0E-A56E-F3E3E5F67FF8; **Taxon:** scientificName: *Mesoveliavittigera* Horváth, 1895; family: Mesoveliidae; genus: Mesovelia; specificEpithet: *vittigera*; scientificNameAuthorship: Horváth, 1895; **Location:** country: Republic of Korea; stateProvince: Chungcheongnam-do; locality: Jangam-ri (36.010707, 126.666094), Janghang-eup, Seocheon-gun; **Identification:** identifiedBy: Seokin Yang & Wonwoong Kim; **Event:** eventDate: 19.vi.2024
- **Type status:** Other material. **Occurrence:** recordedBy: Seokin Yang; individualCount: 2; sex: 2♀; lifeStage: adult; occurrenceID: 65FA577A-09F1-5DEA-B075-DF98390875CC; **Taxon:** scientificName: *Mesoveliavittigera* Horváth, 1895; family: Mesoveliidae; genus: Mesovelia; specificEpithet: *vittigera*; scientificNameAuthorship: Horváth, 1895; **Location:** country: Republic of Korea; stateProvince: Gyeongsangbuk-do; locality: Hahoe-ri (36.537252, 128.527654), Pungcheon-myeon, Andong-si; **Identification:** identifiedBy: Seokin Yang & Wonwoong Kim; **Event:** eventDate: 27.vi.2024
- **Type status:** Other material. **Occurrence:** recordedBy: Seokin Yang; individualCount: 5; sex: 3♂, 2♀; lifeStage: adult; occurrenceID: 615085FA-186F-5AC6-AA87-E23C4EAA64B7; **Taxon:** scientificName: *Mesoveliavittigera* Horváth, 1895; family: Mesoveliidae; genus: Mesovelia; specificEpithet: *vittigera*; scientificNameAuthorship: Horváth, 1895; **Location:** country: Republic of Korea; stateProvince: Jeollanam-do; locality: Ganmun-ri (35.180378, 127.543011), Ganjeon-myeon, Gurye-gun; **Identification:** identifiedBy: Seokin Yang & Wonwoong Kim; **Event:** eventDate: 16.vi.2024

#### Distribution

Widespread in the Palaearctic Region (Andersen 1995). In East Asia: Korea (CN, GG, GB, GN, GW, JJ, JN, Seoul) (Kwon et al. 2001), China (north-western, south-eastern, south-western) (Andersen 1995, Jorigtoo and Qi 1996), Japan (Honshu, Shikoku, Kyushu, Tsushima Island, Nansei Islands) (Hayashi and Miyamoto 2018, Nakajima et al. 2020), Taiwan (Andersen 1995), Russia (Far East: Primorsky Krai) (Kanyukova 1988).

#### Biology

In South Korea, *M.vittigera* inhabited a wide range of freshwater habitats including lakes, reservoirs, rice paddies and brackish ponds. All confirmed habitats were still waterbodies with abundant floating vegetation, some were highly stagnant, densely covered with algal blooms (Fig. 2A).

#### Notes

This species was initially recognised from Korea by Tanaka (1937) as *Mesoveliaorientalis* Kirkaldy, 1901, who subsequently provided distributional information from 淸凉里, 京城 (= currently Cheongnyangni, Seoul) (Tanaka 1939). Miyamoto and Lee (1963) and Miyamoto and Lee (1966) provided further occurrence records from Gyeongsang Province and Jeju island, respectively. Until the mid-1980s, this species was referred to as *M.orientalis* in various Korean literature, despite the early synonymisation with *Mesoveliavittigera* Horváth, 1895 by Horváth (1915). The Ministry of Education (1988) listed both *M.orientalis* and *M.vittigera* and provided a new Korean vernacular name for *M.vittigera*; however, the figure of *M.vittigera* (p. 410, fig. 14) actually depicts a nymph of a *Mesovelia* species, making the identification unstable. Yoon (1995) adopted the same information (p. 111, figs. 4-28 and 29) without modification. In contrast, Lee and Kwon (1991) and Kwon et al. (2001) listed only *M.vittigera*, recognising *M.orientalis* as invalid. This information was followed by later Korean checklists (Lee et al. 2013, Korean Society of Applied Entomology and The Entomological Society of Korea 2021, National Institute of Biological Resources 2023).

Historical distributional records of Mesoveliidae in most Korean literature (except for *M.egorovi* in Lee et al. (1994)) only refer to *M.vittigera* (see Kwon et al. (2001) for bibliographic reference). However, our survey has shown that *M.vittigera* and *M.thermalis* share the same habitat in some affirmed cases and identification of known *Mesovelia* species in Korea requires microscopic observation of both sexes. Therefore, re-examination of previously reported sites of *M.vittigera* in Korea will be critical to clarify the distribution of *Mesovelia* species in South Korea.

### Speovelia maritima

Esaki, 1929

E3BCA4C4-B67A-5138-8E7C-110F5F189DAB

#### Materials

- **Type status:** Other material. **Occurrence:** recordedBy: Seokin Yang & Wonwoong Kim; individualCount: 30; sex: 1♂, 29♀; lifeStage: adult; occurrenceID: 47D5B533-C944-573F-9016-9A536A361926; **Taxon:** scientificName: *Speoveliamaritima* Esaki, 1929; family: Mesoveliidae; genus: Speovelia; specificEpithet: *maritima*; scientificNameAuthorship: Esaki, 1929; **Location:** country: Republic of Korea; stateProvince: Chungcheongnam-do; locality: Sinheuk-dong (36.323476, 126.502176), Boryeong-si; **Identification:** identifiedBy: Seokin Yang & Wonwoong Kim; **Event:** eventDate: 19.vi.2024
- **Type status:** Other material. **Occurrence:** recordedBy: Seokin Yang & Woochan Seo; individualCount: 58; sex: 58♀; lifeStage: adult; occurrenceID: 00AB13E4-9BF0-5B64-81AE-43835A957CD1; **Taxon:** scientificName: *Speoveliamaritima* Esaki, 1929; family: Mesoveliidae; genus: Speovelia; specificEpithet: *maritima*; scientificNameAuthorship: Esaki, 1929; **Location:** country: Republic of Korea; stateProvince: Chungcheongnam-do; locality: Sinheuk-dong (36.323476, 126.502176), Boryeong-si; **Identification:** identifiedBy: Seokin Yang & Wonwoong Kim; **Event:** eventDate: 03.vii.2024

#### Distribution

Korea (CN), Japan (Hokkaido, Honshu, Izu Islands, Ogasawara Islands, Oki Islands, Shikoku, Kyushu, Nansei Islands) (Hayashi and Miyamoto 2018, Nakajima et al. 2020).

#### Biology

According to Esaki (1929) and Yuasa (1929), this species dwells on the walls of moist littoral caves where external lights are completely absent. Several distributional records from various localities along the coastline of Japan have been published since, but detailed information on its life history is scarce (cf. Matsuda (2016)).

We collected a number of specimens on the coast of the Yellow Sea, where sandy beaches meet rocky shores and concrete tetrapods (Fig. 2E). Confirmed habitats were relatively dry and not submerged even in high tides. Large numbers of *S.maritima* were found actively crawling on the concrete tetrapods and rocks at night. Despite diligent searching, no single individual was found active during the daytime. Taken together, our observations imply that *S.maritima* could be nocturnal, hiding inside the littoral caves or rocky clefts during the day. In addition, an imbalance in sex ratio was found during the survey: one male amongst 29 females in the first field collection in June and 58 females in the second collection in July. No single nymph was confirmed in both surveys.

#### Notes

New record for Korea. This species was previously known as a Japanese endemic.

##### Korean vernacular name

"Ba-da-mul-no-lin-jae" referring to the maritime habitat.

## Identification Keys

### Keys to species of Korean Mesoveliidae

**Table:** 

1	Dorsum reddish-brown; length of head significantly longer than width (Fig. 1E and F)	*Speoveliamaritima* Esaki, 1929
–	Dorsum olive-brown to green; length of head subequal to width	2
2	Male sternum VIII with 2 median black spinules; Female sternum VIII with 2 processes at the posterior margin; dorsum green, with dark stripes alongside thoracic and abdominal segments (Fig. 1C and D)	*Mesoveliathermalis* Horváth, 1915
–	Male sternum VIII with single median black spinule; dorsum without dark patterns	3
3	Female sternum VIII without posterior process; dorsum green (Fig. 1A and B)	*Mesoveliavittigera* Horváth, 1895
–	Female sternum VIII with 2 processes at the posterior margin; dorsum brown	*Mesoveliaegorovi* Kanyukova, 1981

This key is applicable to the apterous forms and was based on the previous studies by Kanyukova (1988) and Watanabe et al. (2023).

## Supplementary Material

XML Treatment for Mesovelia egorovi

XML Treatment for Mesovelia thermalis

XML Treatment for Mesovelia vittigera

XML Treatment for Speovelia maritima

## Figures and Tables

**Figure 1.**
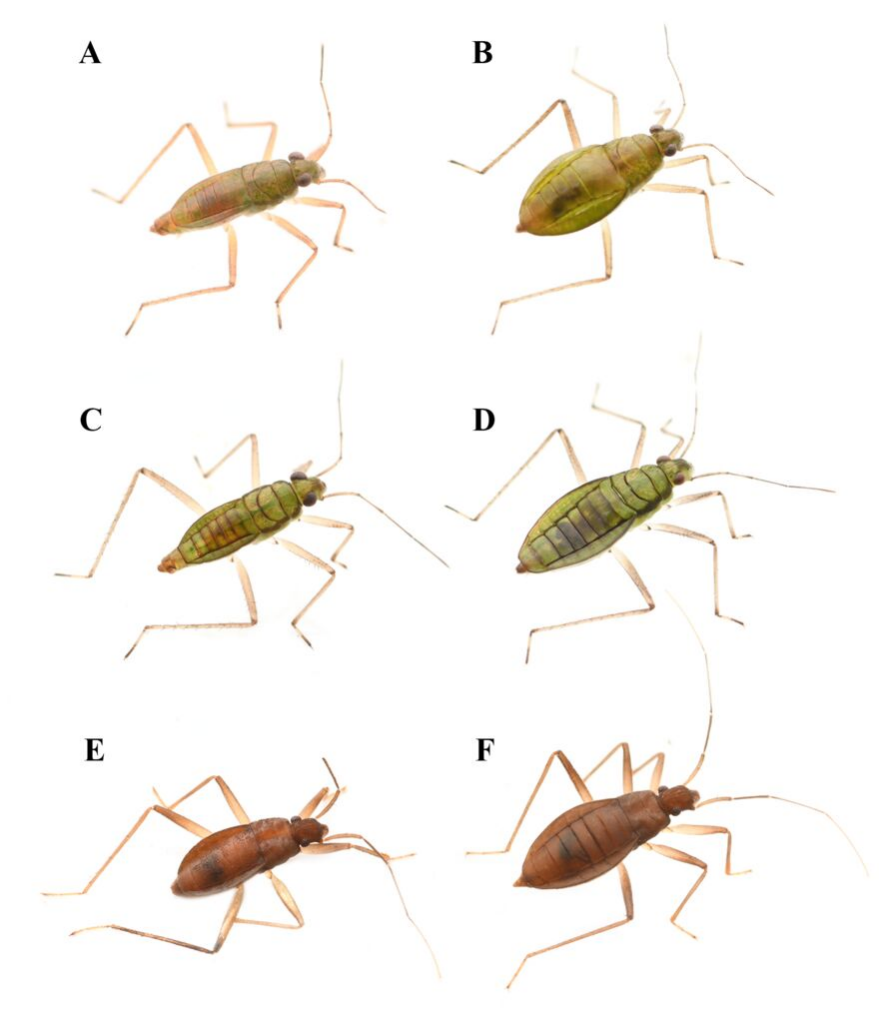
**Dorsal habitus of known Mesoveliidae species of Korea. A** *Mesoveliavittigera* Horváth, 1895, male; **B** ditto, female; **C** *Mesoveliathermalis* Horváth, 1915, male; **D** ditto, female; **E** *Speoveliamaritima* Esaki, 1929, male; **F** ditto, female. Figures not to scale.
